# Pediatric Responses to Fundamental and Formant Frequency Altered Auditory Feedback: A Scoping Review

**DOI:** 10.3389/fnhum.2022.858863

**Published:** 2022-05-17

**Authors:** Caitlin Coughler, Keelia L. Quinn de Launay, David W. Purcell, Janis Oram Cardy, Deryk S. Beal

**Affiliations:** ^1^Graduate Program in Health and Rehabilitation Sciences, Faculty of Health Sciences, The University of Western Ontario, London, ON, Canada; ^2^Bloorview Research Institute, Holland Bloorview Kids Rehabilitation Hospital, Toronto, ON, Canada; ^3^Rehabilitation Sciences Institute, Temerty Faculty of Medicine, University of Toronto, Toronto, ON, Canada; ^4^School of Communication Sciences and Disorders, Faculty of Health Sciences, The University of Western Ontario, London, ON, Canada; ^5^National Centre for Audiology, Faculty of Health Sciences, The University of Western Ontario, London, ON, Canada; ^6^Department of Speech-Language Pathology, Temerty Faculty of Medicine, University of Toronto, Toronto, ON, Canada

**Keywords:** altered auditory feedback, speech motor control, sensorimotor learning, speech development, fundamental frequency manipulation, formant frequency manipulation

## Abstract

**Purpose:**

The ability to hear ourselves speak has been shown to play an important role in the development and maintenance of fluent and coherent speech. Despite this, little is known about the developing speech motor control system throughout childhood, in particular if and how vocal and articulatory control may differ throughout development. A scoping review was undertaken to identify and describe the full range of studies investigating responses to frequency altered auditory feedback in pediatric populations and their contributions to our understanding of the development of auditory feedback control and sensorimotor learning in childhood and adolescence.

**Method:**

Relevant studies were identified through a comprehensive search strategy of six academic databases for studies that included (a) real-time perturbation of frequency in auditory input, (b) an analysis of immediate effects on speech, and (c) participants aged 18 years or younger.

**Results:**

Twenty-three articles met inclusion criteria. Across studies, there was a wide variety of designs, outcomes and measures used. Manipulations included fundamental frequency (9 studies), formant frequency (12), frequency centroid of fricatives (1), and both fundamental and formant frequencies (1). Study designs included contrasts across childhood, between children and adults, and between typical, pediatric clinical and adult populations. Measures primarily explored acoustic properties of speech responses (latency, magnitude, and variability). Some studies additionally examined the association of these acoustic responses with clinical measures (e.g., stuttering severity and reading ability), and neural measures using electrophysiology and magnetic resonance imaging.

**Conclusion:**

Findings indicated that children above 4 years generally compensated in the opposite direction of the manipulation, however, in several cases not as effectively as adults. Overall, results varied greatly due to the broad range of manipulations and designs used, making generalization challenging. Differences found between age groups in the features of the compensatory vocal responses, latency of responses, vocal variability and perceptual abilities, suggest that maturational changes may be occurring in the speech motor control system, affecting the extent to which auditory feedback is used to modify internal sensorimotor representations. Varied findings suggest vocal control develops prior to articulatory control. Future studies with multiple outcome measures, manipulations, and more expansive age ranges are needed to elucidate findings.

## Introduction

The ability to produce speech begins shortly after birth as infants begin mapping speech sounds onto the position and movement of articulators during the babbling stage (Siegel et al., 1976; de Boysson-Bardies, 2001; Civier et al., 2010). By 3 years of age, children can speak fluently, mastering a variety of consonant and vowel sounds to form words (Coplan and Gleason, 1988). During this early development, dramatic anatomical changes occur to the shape, size, and muscles of the structures involved in speech production (Guenther, 1994; Kent and Vorperian, 1995; Kent, 1999; Callan et al., 2000). Despite these changes, children’s speech remains relatively fluent through the support of speech motor control (Guenther, 1994; Callan et al., 2000). Motor actions involved in speech production are monitored, and execution errors are detected and subsequently corrected, through feedback and feedforward mechanisms (Guenther, 2006; Alsius et al., 2013). Feedback controllers use sensory information (i.e., auditory and somatosensory feedback) to monitor and adjust motor commands sent to speech production articulators (i.e., vocal tract and larynx). Feedforward controllers guide the production of motor commands by reading out previously learned motor programs, without using incoming sensory information. Speech production requires both feedback and feedforward control, with auditory feedback playing a key role.

Auditory feedback, that is, our ability to hear ourselves speak, has been shown to play an important role in the development and maintenance of intelligible speech *via* studies showing how speech acquisition is negatively impacted when hearing is impaired at birth (Oller and Eilers, 1988), as well as how speech deteriorates following loss of hearing later in life (Cowie and Douglas-Cowie, 1992). As auditory feedback informs our correct production of speech, analyzing children’s speech production under altered auditory feedback can provide important information about how auditory feedback is involved in the maturing speech motor control system. In the present scoping review, the use of frequency altered auditory feedback, specifically fundamental and formant frequency manipulations, in speech production research with pediatric populations was examined. Responses to these manipulations provide key information about articulatory and vocal motor control.

### Altered Auditory Feedback Paradigms

Altered auditory feedback paradigms have been used to study auditory processing, sensorimotor control, and auditory-motor integration, independent of factors such as memory, complexity, or attentional control. This paradigm has been used in adults to expand our understanding of auditory feedback. Auditory feedback plays an important role in two primary functions: (a) accommodating *vocal settings* of respiratory, laryngeal, and supraglottal systems, and (b) maintaining *articulatory settings* to preserve phonemic distinctions and intelligibility (Perkell et al., 1997; Möbius and Dogil, 2002). Fundamental frequency (*f*_0_), whose perceptual correlate is vocal pitch, is associated with vocal control. Fundamental frequency relates to the positioning and frequency of vocal fold vibrations and is determined by the length and tension of the vocal folds (Stemple et al., 2000; Zhang, 2016). Shifted fundamental frequency results in participants hearing their own voice sound higher or lower in pitch than anticipated. Formant frequencies relate to the positioning of the lip, tongue, and jaw, or our articulation, with changes in formant frequencies resulting in different sounds (and words) being produced (Anstis and Cavanagh, 1979; Elman, 1981; Larson, 1998; Purcell and Munhall, 2006b). The first formant (F1) is inversely related to tongue height, where sounds with a higher tongue position have a lower F1. The second formant (F2) is related to tongue front or backness, where sounds closer to the front of the mouth (e.g., lips) have a higher F2. Formant frequency manipulation studies aim to shift F1 and/or F2 and measure the participants’ responses. For example, if the F1 in the vowel/ε/is raised by approximately 200 Hz in the word “head,” the auditory feedback provided to the talker would be closer to that of the word “had” with the vowel/ae/. Manipulations of speech sounds characteristics other than vowel formants have also been used to examine articulatory control. For example, a change in the first spectral moment, or frequency centroid, of sibilant fricatives (e.g., /s/) results in participants perceiving a shifted version of the fricative (e.g., closer to/∫/or “sh”). The effects of altering auditory feedback of fundamental and formant frequencies has been extensively studied in adults using altered auditory feedback paradigms where these acoustic parameters are shifted in real-time and the magnitude, direction, timing and variability of compensatory responses to these shifts are studied (Burnett et al., 1998, 1997; Houde and Jordan, 1998). These responses have been examined in paradigms of *unexpected* trial shifts and predictable *sustained* shifts.

#### Unexpected Shift

Altered auditory feedback studies using sudden, unexpected shifts have explored how participants respond when their auditory feedback is shifted multiple times during a sustained vocalization (Behroozmand et al., 2009), at a random point during sustained vocalizations (Larson et al., 2001; Franken et al., 2018), or during a random trial (Elman, 1981; Burnett et al., 1997; Purcell and Munhall, 2006a; Tourville et al., 2008). Participants in *f*_0_ and formant manipulated auditory feedback studies have been found to typically produce a *reflexive compensatory* response in the opposite direction of the manipulation (Burnett et al., 1997; Hain et al., 2000). These responses are usually only partial compensations to the shift (Purcell and Munhall, 2006a; Chen et al., 2007). Although manipulations in *f*_0_ studies typically range from ±25 to 600 cents (100 cents = 1 semitone), participants on average produce response magnitudes of less than 60 cents (Burnett et al., 1997; Larson et al., 2000; Natke and Kalveram, 2001; Burnett and Larson, 2002; Donath et al., 2002; Bauer and Larson, 2003; Natke et al., 2003; Sivasankar et al., 2005; Liu and Larson, 2007). In formant manipulation studies, participants produce compensatory responses that are on average less than 30% of the total shift (Purcell and Munhall, 2006a; Tourville et al., 2008; Mitsuya et al., 2015). A second response type, where vocal productions follow the same direction as the shift, called *following* responses, has also been observed (Burnett et al., 1997, 1998; Hain et al., 2000; Larson et al., 2007). It has been suggested that these following responses occur more frequently with large magnitude shifts (i.e., in *f*_0_ perturbations; Burnett et al., 1998). Behroozmand et al. (2012) and Franken et al. (2018) however found that most individuals who produced opposing (compensatory) responses on average, tended to also produce following responses on some trials.

Both following and compensatory responses typically show an onset latency of approximately 100–150 ms, suggesting these responses are reflexive and automatic (Tourville et al., 2008). This has been supported by findings showing that participants produce compensatory productions even when instructed to ignore any manipulations (Burnett et al., 1997; Zarate and Zatorre, 2008; Patel et al., 2014; Hu et al., 2015). Hain et al. (2000), however, found that there appear to be two responses produced in *f*_0_ manipulations: an early automatic response that can be modulated by instruction and a later response under voluntary control. Overall, these compensatory responses are thought to provide information about an individual’s auditory feedback control (Tourville et al., 2008; Cai et al., 2011). Larger response magnitudes opposing the direction of the shift are postulated to reflect greater reliance on auditory feedback, although the magnitude and direction of the applied perturbation in studies need to be taken into consideration (Heller Murray and Stepp, 2020).

#### Predictable Sustained Shift

In contrast, predictable, *sustained* auditory shifts are used to evaluate the updating of the feedforward system (feedforward control) through sensorimotor adaptation. In these paradigms, participants are presented with shifted auditory feedback stimuli over multiple successive trials, and gradually develop/learn an *adaptive response* to compensate for the perturbation (Houde and Jordan, 1998, 2002; Jones and Munhall, 2000, 2002; Purcell and Munhall, 2006b; Villacorta et al., 2007). In adults, these compensatory effects remain immediately following removal of the altered auditory feedback; such *adaptation* indicates a learned response in which stored motor programs have been updated (adapted) in response to the persistent compensatory productions made (Houde and Jordan, 1998, 2002; Jones and Munhall, 2000; Purcell and Munhall, 2006b). These studies typically consist of four phases: a baseline phase, where participants receive normal feedback; followed by a ramp phase, where the auditory feedback is incrementally shifted; then a hold phase, where the shifted stimuli is held at its maximum; and finally, sometimes, an end phase where the perturbations are removed. In these studies, two responses are examined: (a) how individuals’ responses during the hold phase differ from their average baseline phase productions (looking for *compensation* to shifts), and (b) how individuals’ productions at the end phase (when the perturbation is removed) differ from the baseline phase (looking for *adaptation*).

These adaptation paradigms provide key information about how speakers use auditory feedback, for calibration and maintenance (i.e., during hold phase) and to incorporate long-term changes in their speech production (i.e., during end phase; Houde and Jordan, 1998; Purcell and Munhall, 2006b; MacDonald et al., 2010). Similar to the reflexive responses to sudden perturbations, participants’ responses are typically in the opposite direction of the manipulation, with some responses following the perturbations (Houde and Jordan, 1998, 2002; Jones and Munhall, 2000, 2005; Purcell and Munhall, 2006b; Villacorta et al., 2007). These responses also only partially compensate for the total perturbation magnitude (Jones and Munhall, 2000, 2005; Houde and Jordan, 2002; Purcell and Munhall, 2006b; Villacorta et al., 2007; MacDonald et al., 2010). Katseff et al. (2012) suggested that based on findings that individuals showed greater compensation for small F1 perturbations than for larger perturbations, auditory feedback may play a larger role in small discrepancies. These responses have also been found to be automatic, occurring when participants are instructed to ignore manipulations (Munhall et al., 2009; Keough et al., 2013).

Across studies exploring responses to sudden shifts, there is consensus that these responses describe *compensation*. However, within the sensorimotor adaptation literature, inconsistencies persist. In some studies, responses produced when the perturbation is held at its maximum (hold phase trials) are described using the term *compensation*, while in others, these trials are referred to as *adaptation.* Although responses in these trials are thought to gradually reflect updating of motor commands and hence adaptation to the shift, within this article, the term *compensation* will be used to describe responses within the hold phase of a sensorimotor adaptation paradigm, as these productions represent both compensation and adaptation. Productions made following removal of shifts (during end phase trials) will be described as *adaptation* (also known in the literature as after-effects).

#### Relations Between Unexpected and Sustained Perturbation

Examining responses to unexpected and sustained perturbations provides important information about feedback and feedforward control. Contrasting participants’ responses to sudden and sustained F1 manipulations, Franken et al. (2019) and Raharjo et al. (2021) found that individuals’ responses in the sudden vs. sustained conditions were not correlated with each other. In contrast, Lester-Smith et al. (2020) explored reflexive compensatory and adaptive responses to F1 and *f*_0_ perturbation. Participants showed similarities in their reflexive and adaptive responses, where individuals with larger reflexive responses to sudden F1 perturbation also showed larger adaptive responses to predictable F1 manipulations. However, reflexive and adaptive responses to *f*_0_ manipulated auditory feedback were not related. This highlights that differences may not only be evident in the mechanisms underlying responses in sudden (reflexive) and sustained (adaptive) perturbation paradigms, but also in control of articulatory and vocal settings. Although responses to fundamental and formant frequency altered auditory feedback have extensively been studied in adults, a contrastive look at responses in children has not previously been examined. Investigating how and if these responses differ developmentally will help shed light on underlying mechanisms and improve our understanding of speech motor control.

### Models of Speech Motor Control

Prominent models of speech motor control have strived to model how we regulate our speech production. The *directions into velocities of articulators* (DIVA) model uses auditory and somatosensory feedback control combined with feedforward control to maintain fluent speech (Guenther, 1995, 2016; Guenther et al., 1998; Tourville and Guenther, 2011). Mismatches in the feedback control systems between the actual and expected sensory state are used to form corrective motor commands (Tourville and Guenther, 2011; Guenther and Vladusich, 2012). In a sustained shift condition, over multiple corrective motor commands, these adjustments are used to update the feedforward command. In this way, the DIVA model postulates that similar mechanisms are employed in response to sudden and sustained perturbations. In contrast, the *state feedback control* (SFC) model postulates that responses to sudden and sustained perturbations are driven by different mechanisms. In the SFC model, sensory feedback can be used directly to update the internal model estimate of the dynamical state of the vocal tract (Houde and Nagarajan, 2011; Houde et al., 2013). Thus, unlike the DIVA model, the SFC model does not require the integration of corrective motor commands into feedforward control in order to accommodate adaptation.

### Neurophysiology and Neuroimaging Association

Behavioral data from altered auditory feedback paradigms provide information about the final product of the manipulation, the vocal response to the shift. This data however, does not elucidate what may be contributing to differences in these responses. Examining neural activation and neural structure provide key information about how the brain processes stimuli leading to the final vocal production. Neurophysiology (e.g., EEG) and neuroimaging (e.g., MRI) are useful in conjunction with altered auditory feedback paradigms, however, it is important to take into consideration potential limitations of these techniques. While a comprehensive review of potential caveats that might hamper the interpretability of these techniques is out of the scope for this article, one of the biggest challenges to consider when using these techniques with altered auditory feedback is filtering out activation that occurs as a result of motor movement from spoken productions.

Neurophysiological and neuroimaging data have been instrumental in informing models of speech motor control. Using neuroimaging (i.e., functional magnetic resonance imaging [fMRI]), individual components of the DIVA model have been mapped onto brain regions based on experimental neuroimaging findings (Bohland and Guenther, 2006; Ghosh et al., 2008; Tourville et al., 2008; Golfinopoulos et al., 2011). Examining typical neural regions of activation in response to altered auditory feedback (i.e., using fMRI), as well as structural similarities (i.e., using diffusion-weighted imaging), provides important information relating to typical and atypical productions, expanding our understanding of neural correlates related to feedback and feedforward control. While MRI provides excellent spatial resolution, it has low temporal resolution as it measures changes in blood flow.

Electroencephalography (EEG) in contrast has high temporal resolution and low spatial resolution, making it an ideal methodology to examine the timing of neural responses, which is particularly important given the quick pace of speech. Neurophysiological activity measured through EEG responses to auditory stimuli provides important information about the processing of auditory input, expanding on behavioral findings (Hillyard and Picton, 1978). Common event related potentials observed in response to auditory stimuli are characterized by a positive-negative-positive sequence, the P1-N1-P2 complex. The initial positive peak (P1) is approximately 30–110 ms after stimulus onset, followed by a negative peak (N1) approximately 80–150 ms after stimulus onset, and a final positive peak (P2) 140–160 ms after stimulus onset (Ponton et al., 2000). Developmentally, latency of the P1 and N1 components has been found to negatively correlate with age in response to speech and non-speech stimuli (Polich et al., 1990; Kraus et al., 1993; Tonnquist-Uhlen et al., 1995; Cunningham et al., 2000; Ponton et al., 2000; Wunderlich and Cone-Wesson, 2006), whereas the latency of the P2 component has not been found to significantly vary with age (Ponton et al., 2000; Fitzroy et al., 2015). In terms of amplitude, P1 has been found to decrease with age in response to speech and non-speech stimuli (Kraus et al., 1993; Sharma et al., 1997; Cunningham et al., 2000; Fitzroy et al., 2015), whereas N1 and P2 amplitudes are less consistently found to change developmentally (Sharma et al., 1997; Wunderlich and Cone-Wesson, 2006; Fitzroy et al., 2015).

During EEG altered auditory feedback studies in adults, increased activity has been found in the P1-N1-P2 complex (Heinks-Maldonado et al., 2006; Behroozmand et al., 2011). Amplitudes of the N1 and P2 components have been found to be correlated with the magnitude of perturbations, whereas the amplitude of the P1 component has been found to increase in a non-specific manner (Liu et al., 2011; Scheerer et al., 2013a). Based on these patterns of response, it has been theorized that P1 represents a general recognition of a mismatch between expected and actual auditory feedback, whereas N1 is related to the determination of whether feedback is internally or externally generated, and P2 represents processing of the size of the mismatch (Scheerer et al., 2013a). As the P1-N1-P2 complex has been associated with age-related changes during auditory processing, exploring differences in neurophysiological activity during altered auditory feedback paradigms provides an additional avenue for expanding our understanding of developmental differences in the use of auditory feedback.

### Speech, Language, Auditory Feedback, and Clinical Populations

Speech and language processes are interactive and influence each other throughout development, and examining their interaction can provide key developmental information (Kent, 2004; Smith and Goffman, 2004; Terband and Maassen, 2010; Strand et al., 2013). Reading, in turn, builds on these speech and language skills (Mattingly, 1972; Liberman, 1989; Rueckl et al., 2015). As such, speech motor control impairments have been documented in children with speech sound disorders (SSD; Namasivayam et al., 2013), individuals who stutter (Bloodstein and Bernstein-Ratner, 2008), individuals with dyslexia (van den Bunt et al., 2017), and children with developmental disorders that often include co-occurring language impairments such as autism spectrum disorder (ASD; Belmonte et al., 2013). Exploring the differences in the integration of auditory information during speech in children with speech and language disorders could provide more insight into the mechanisms that typically developing children use to respond and process this feedback.

While auditory feedback is considered important for the development of speech motor control, the means by which children use this feedback to establish and refine their internal sensorimotor representations and to control online speech production remain relatively unknown. Specifically, determining children’s capacity to integrate auditory information into upcoming motor commands is essential to better understanding the role of auditory feedback in the acquisition and refinement of speech production, as well as the mechanisms that govern compensation for changes in auditory feedback throughout development. The purpose of this scoping review is to explore the current use of frequency altered auditory feedback paradigms in pediatric speech research, and investigate how the integration of auditory information during speech changes across development, through an examination of behavioral responses to auditory perturbation in pediatric populations. This is essential for ultimately understanding the mechanisms underlying the acquisition of speech motor control.

## Methods

### Objectives and Rationale

A scoping review of published studies was conducted to identify existing articles that have used frequency altered auditory feedback paradigms with children. The structured framework presented by Arksey and O’Malley (2005) and further developed by Levac et al. (2010) was utilized. The aim of our scoping review was to identify and analyze the current state of research for pediatric responses to frequency altered auditory feedback in order to examine how the research has been conducted, clarify key concepts, summarize the current evidence, and identify gaps in the existing research. Research questions included: (a) what were the characteristics of the children included in these studies (e.g., age ranges, clinical populations, country, language); (b) how many studies included a manipulation of fundamental frequency, formant frequency, or both; (c) are differences in responses to altered auditory feedback evident across developmental stages and clinical populations, and (d) what methodological designs have been used with children?

### Search Strategy

The literature was searched for publications up until October 19th, 2021. Searches were conducted in six academic databases: CINAHL, Embase, Medline (*via* Ovid), PsycINFO (*via* Proquest), Scopus, and Web of Science. A standardized list of keywords, as well as database subject headings (MeSH) for the concepts of altered auditory feedback and pediatric were developed (see Supplementary Material 1). All six databases were searched using keywords. MeSH searches were combined with their respective keyword searches (altered auditory feedback or pediatric) for four databases (CINAHL, Embase, Medline and PsycInfo). Supplementary Material 2 shows a sample of how the search was conducted in PsycInfo. Following searches of all databases, citations were uploaded to the referencing software, Covidence (Covidence Systematic Review Software, 2020).

### Inclusion Criteria

**FIGURE 1:**
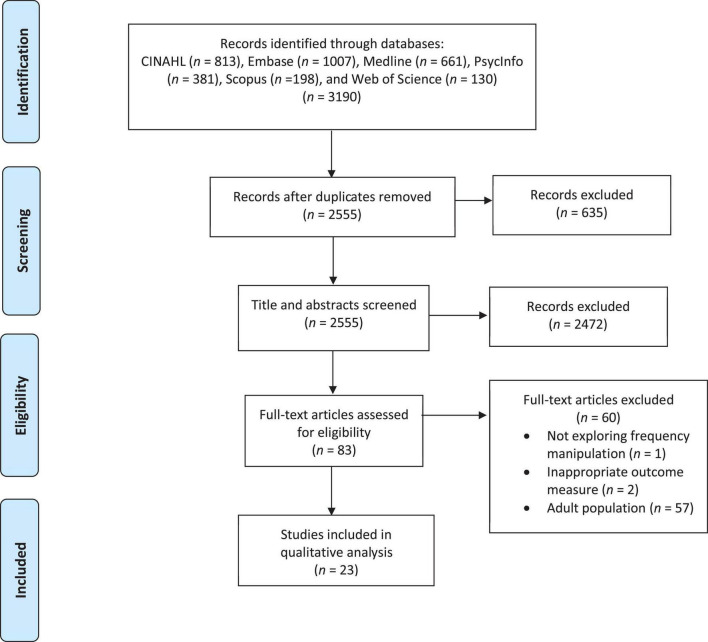
PRISMA diagram of the systematic review of articles for inclusion.

A PRISMA flow chart showing the systematic selection of articles for inclusion is provided in Figure 1. Titles and abstracts were screened for inclusion in the full-article review using three criteria: (a) real-time perturbation of frequency auditory input was used (i.e., formant or fundamental frequency), (b) an analysis of immediate effects on speech in response to the perturbation (e.g., compensatory response) was included, and (c) typically developing (TD) and/or clinical participants between the ages of 2–18 years were included in the sample. Articles that were not experimental studies (e.g., commentaries) were excluded, with the exception of one review article that was included because it introduced a relevant case study. Titles and abstracts were additionally screened by an independent graduate student to ensure no selection bias was present. Inter-rater reliability showed 97% agreement, with a Cohen’s Kappa of 0.62, showing substantial agreement. During full-text review, articles that did not separate participants under 18 years old from adults in the analysis were additionally excluded (*n* = 57). Articles were also excluded due to not exploring frequency manipulated altered auditory feedback (*n* = 1) or analyzing compensatory and/or adaptation responses (*n* = 2).

### Data Extraction

Complete records of data extracted from all articles can be found in Supplementary Material 3.^1^ Information extracted included: (a) article information (title, authorship, year, country conducted in), (b) participant characteristics (age range, sample size, language spoken), (c) primary aim of study, (d) speech and language measures collected, (e) perturbation information (e.g., pitch or formant manipulation, and direction and magnitude), (f) methodological design (e.g., number of trials in each phase), and (g) main outcomes.

## Findings

### Search Outcome

Twenty-three articles were identified as meeting inclusion criteria across the six databases. Of these articles, manipulations included: nine of fundamental frequency, twelve of formant frequency, one of the frequency centroid of fricatives (henceforth grouped as formant manipulation), and one of both fundamental and formant frequencies. Of these studies, nine looked at clinical populations in addition to TD children (three *f*_0_, seven formant^2^). All included studies examined behavioral responses to altered auditory feedback, with four also exploring neurophysiological responses (two electrophysiological, one diffusion-weighted imaging, and one resting-state functional connectivity). See Supplementary Material 3 (see Footnote 1) for an in-depth description of our findings.

### Research Context and Participant Characteristics

The majority of the studies reviewed were conducted in Canada (ten total; four *f*_0_ manipulation, six formant manipulation), followed by the United States (seven total [see Footnote 2]; four *f*_0_, four formant), the Netherlands (three formant), and China (two *f*_0_), and one study which collected children in the United States and the Netherlands (formant). The majority of participants were English speakers (16 total [see Footnote 2]; eight *f*_0_ and nine formant), followed by Dutch (three formant), Mandarin Chinese (two *f*_0_), and French (one formant). All studies included pediatric populations under 18 years with the exception of one clinical population group that included individuals up to 18.3 years of age (as well as TD children under 18 years). Six *f*_0_ manipulation studies and seven formant manipulation studies also included adult populations.

### Methodological Designs and Findings

Methodological designs used with children were divided into those relating to vocal control (i.e., *f*_0_ manipulation) and those relating to articulatory control (i.e., formant manipulations) in order to explore potential influences of these differing paradigms on response outcomes, as well as potentially differing developmental trajectories.

#### Fundamental Frequency Manipulation

All ten of the studies exploring responses to *f*_0_ altered auditory feedback involved unexpected (within trial) perturbations. Two studies also contrasted responses to unexpected perturbations with sustained (predictable) manipulations (Scheerer et al., 2016; Heller Murray and Stepp, 2020). In terms of manipulations applied, all ten of the studies included a negative manipulation of one semitone (−100 cents). Five of the studies also included a positive manipulation of one semitone (+100 cents), and two studies included additional magnitude manipulations either in negative (−50, −200 cents; Liu et al., 2013) or negative and positive directions (±50, 200, and 500 cents; Liu P. et al., 2010). Table 1 includes a summary of findings, sample size, age range and manipulations of *f*_0_ manipulation studies.

**TABLE 1:** Behavioral findings in response to fundamental frequency (f_0_) manipulated altered auditory feedback in typically developing (TD) children and children with speech and language disorders.

Study	Child sample size	Child age range	Manipulation	Findings	Adult contrast	RL	VV	CF	NF
Scheerer et al. (2020a)	(1) *n* = 11 (2) *n* = 9	(1) 24–35 months (2) 40–46 months	“baa” Within trial ±100 cents	• Both groups of toddlers compensated to the perturbation • No main effect of age found			X		
Scheerer et al. (2016)	*n* = 25	3.0–8.0 years	/a/ (1) Within trial (unpredictable) −100 cents (2) Sustained (predictable) −100 cents	• Children showed compensation in both designs but *smaller responses* than adults • Children *more variable*	X		X		
Scheerer et al. (2020b)	(1) *n* = 45 (2) *n* = 30*	3.0–13 years	/a/ Within trial ±100 cents	• Autistic children had *shorter response latencies* • Both autistic and TD children compensated in opposite direction of shift (similar in magnitude and variability)			X	X	
Scheerer et al. (2013b)	*n* = 80 children (10 M, 10 F per group)	(1) 4–6 years (2) 7–10 years (3) 11–13 years (4) 14–17 years	/a/ Within trial −100 cents	• Younger children had *longer response latencies* • Children 4–6 years *more variable* than adults • No significant interaction of age and sex on response magnitude	X	X	X		X
Heller Murray and Stepp (2020)	*n* = 20	6.6–11.7 years	/α/ (1) Within trial shift ±1 ST (2) Sustained shift ±1 ST	(1) Opposing responses only: children with less sensitive pitch discrimination (C-L; >2 *SD* from adults) showed significantly *larger* responses than adults or children with adult-like pitch discrimination (C-A) (2) C-L had *smaller* vocal response magnitudes than C-A and adults	X		X		
Liu P. et al. (2010)	*n* = 19	7.0–12.0 years	/u/Within trial ±50, 100, 200, 500 cents	• Children showed significantly *larger* compensatory responses to adults • Children produced *longer latencies* than adults	X	X			
Liu H. et al. (2010)	*n* = 10	7.0–12.0 years	/a/ Within trial −100 cents	• Older adults produced significantly larger response magnitudes than children and young adults • Children produced significantly *longer latencies* than younger and older adult groups	X	X			
Russo et al. (2008)	(1) *n* = 19 (2) *n* = 18*	(1) 7.0–12.0 years (2) 7.0–12.0*	/a/ Within trial −100 cents	Subset of children with ASD produced larger responses than TD children			X	X	
Liu et al. (2013)	(1) *n* = 22 (2) *n* = 20	(1) 10–12 years (2) 13–15 years	/u/ Within trial −50, −100, or −200 cents	Younger children elicited *longer latency* vocal response than young adults	X	X			X
Demopoulos et al. (2018)	(1) *n* = 11 (2) *n* = 12**	(1) 10.3–15.4 years (2) 8.3–18.3** years	“ah” Within trial ±100 cents	Children with 16p11.2 deletion showed larger pitch compensation compared to controls				X	

*Refers to children with Autism Spectrum Disorder (ASD), and

**refers to children with 16p11.2 deletion.

*RL, response latencies; VV, vocal variability; CF, clinical findings; NF, neuroimaging findings.*

#### Formant Frequency Manipulation

All fourteen studies exploring responses to formant manipulated auditory feedback explored responses to sustained perturbations over several trials. Table 2 includes a summary of findings, sample size, age range, and formant manipulations, with more in-depth findings available in Supplementary Material 3 (see Footnote 1). Generally, studies included a baseline phase (ranging from 10–50 trials, *M* = 23.0) followed by a ramp phase where the perturbation was gradually introduced (ranging from 10–60 trials, *M* = 25.4), and a hold phase where the perturbation was held at its maximum (ranging from 18–120 trials, *M* = 41.0). Ten studies also included an end phase where the perturbation was removed (ranging from 10–40 trials, *M* = 20.15). One study additionally included a ramp-down phase where the perturbation was gradually removed following the hold phase (van den Bunt et al., 2018b).

**TABLE 2:** Behavioral findings in response to formant manipulated altered auditory feedback in typically developing (TD) children and children with speech and language disorders.

Study	Child sample size	Child age range	Manipulation	Findings	Adaptation	Adult contrast	VV	CF	NF
MacDonald et al. (2012)	(1) *n* = 20 (2) *n* = 26	(1) 23–35 months (2) 43–59 months	/ε/ F1 increased by 200 Hz and F2 decreased by 250 Hz	• Young children compensated in opposite direction of perturbation, but toddlers did not • No significant difference in compensation in adults and young children • Variability decreased with age		X	X		
Kim et al. (2020)	(1) *n* = 8 (2) *n* = 8 (3) *n* = 8^a^ (4) *n* = 8^a^	(1) 3.75–6.83 years (2) 7.25–9.33 years (3) 3.50–6.83^a^ years (4) 7.08–9.33^a^ years	“buck,” “bus,” “puck,” “pup,” “cut,” “cup,” “gut,”, “duck” Upward shift of 335 cents (gradual with ramp and without ramp conditions)	• TD children had similar compensation to TD adults • Both younger and older children who stutter did not show compensation (in either condition)		X		X	
Terband et al. (2014)	(1) *n* = 17 (2) *n* = 11^b^	(1) 4.1–8.7 years (2) 3.9–7.5^b^ years	/ɪː/ F1 increased by 25% and F2 decreased by 12.5%	• Children with SSD followed the perturbation in F1 during hold and end phase • TD children compensated in F1 and F2 and showed trend of adaptation in F1 in end phase	X			X	
Caudrelier et al. (2019)	(1) *n* = 29 (2) *n* = 24	(1) 4–5 years (2) 7–8 years	/e/ F1 increased by 27% and F2 decreased by 10%	• Some preschoolers and school-aged children compensated for the perturbation • No significant difference between groups • Adaptation magnitude similar across age groups	X	X			
van Brenk and Terband (2020)	*n* = 23	4.0–8.7 years	/ɪː/ F1 increased by 25% and F2 increased by 12.5%	• In F1, children showed stronger compensation and adaptation response than adults • In F2, children showed a compensation but no adaptation response • In F1 and F2, children showed higher token-to-token variability than adults	X	X	X		
Shiller and Rochon (2014)	*n* = 22 (Exp and Sham groups)	5.0–7.0 years	/ε/ F1 increased by 25%	• Both Exp and Sham group compensated to perturbation • Following perceptual training, Exp group showed increased magnitude compensation (Sham group showed no change) • Change in F1 persisted after removal of manipulation	X				
van den Bunt et al. (2018a)	US: *n* = 96 NL: *n* = 148	preschool – grade 2 (∼5–8 years)	/ε/ F1 increased by 25%	Significantly stronger compensation in hold and end phase for literate children relative to preliterate children	X		X		
Ohashi and Ostry (2021)	*n* = 19	5–12 years	/ε/ F1 increased to make/ae/average 23.9 ± 1.59% (SE)	Children showed similar compensation to adults, adaptation in children remained longer than adults	X	X	X		X
Coughler et al. (2021)	(1) *n* = 16 (2) *n* = 16^c^	(1) 6.83–11.68 years (2) 7.83–13.2 years	/ε/ (1) F1 increased by 340 Hz (2) F1 decreased by 230 Hz	Children with DLD showed greater compensation in the positive F1 manipulation condition and compensated less than TD children in the negative shift condition	X		X	X	
Shiller et al. (2010b)	*n* = 1^b^	6.5^b^ years	/ε/ F1 increased by 175 Hz	• Compensated to perturbation • Adaptation seen following removal of manipulation	X			X	
Daliri et al. (2018)	(1) *n* = 20 (2) *n* = 20^a^	(1) 7.08–11.42 years (2) 6.08–11.17^a^ years	/ε/ F1 increased by 25% and F2 decreased by 12.5%	• Both children groups compensated to F1 perturbation but not F2 perturbation • No significant difference between adults and children who do not stutter for F1 or F2 perturbation • Children who stutter compensated more than adults who stutter for F1 perturbation	X	X		X	
Shiller et al. (2010a)	*n* = 11	9.4–11.3 years	/s/ frequency centroid decreased by 3 semitones (averaging −1222 Hz)	Children showed compensatory response of similar magnitude to adults (no significant difference)		X	X		
van den Bunt et al. (2018b)	(1) *n* = 10 (2) *n* = 27^d^	10.0–13.0 years	F2-F1 manipulation individualized: from/√/to/ε/at maximum perturbation	• All participants compensated in opposite direction of manipulation • Children with dyslexia showed weaker return to baseline during ramp-down phase than typically reading children	X			X	X
Demopoulos et al. (2018)	(1) *n* = 11 (2) *n* = 12*^e^*	(1) 10.3–15.4 years (2) 8.3–18.3*^d^* years	/ε/ F1 increased by 200 Hz and F2 decreased by 250 Hz	Control children showed significantly greater compensation than children who were 16p11.2 deletion carriers				X	

*^a^Refers to children who stutter.*

*^b^Refers to children with a speech sound disorder (SSD).*

*^c^Refers to children with developmental language disorder (DLD).*

*^d^Refers to children with dyslexia.*

*^e^Refers to children with 16p11.2 deletion.*

*VV, vocal variability; CF, clinical findings; NF, neuroimaging findings.*

In terms of magnitude and direction of manipulations, eight studies manipulated F1 and F2 values of vowels, and five studies manipulated F1 only. F1 was manipulated in various ways, including: increased by 25% (Shiller and Rochon, 2014; van den Bunt et al., 2018a), 175 Hz (Shiller et al., 2010b), or 340 Hz, or decreased by 230 Hz (Coughler et al., 2021), or manipulated individually so the maximum perturbation represented a change from/ε/to/ae/ (Ohashi and Ostry, 2021). One study manipulated the frequency centroid of fricatives (decreased by 3 semitones; Shiller et al., 2010a). Manipulations of F1 and F2 were language dependent. In Dutch, this manipulation included an F1 increase of 25% and an F2 increase or decrease by 12.5% (Terband et al., 2014; van Brenk and Terband, 2020 respectively), in French F1 was increased by 27% and F2 decreased by 10% (Caudrelier et al., 2019), and in English F1 was increased by 200 Hz or 25% and F2 was decreased by 250 Hz or 12.5% (MacDonald et al., 2012; Daliri et al., 2018; Demopoulos et al., 2018). One study (van den Bunt et al., 2018b) individualized the manipulation so the maximum perturbation meant a change from/ı/to/ε/for each participant. Kim et al. (2020) manipulated F1 and F2 upward 335 cents (adult population received manipulation of ±250 cents).

Results across both fundamental and formant frequency manipulations found age-dependent developmental trajectories related to response latencies, magnitude of compensatory responses, variability and perceptual abilities, as well as relationships of compensation with literacy abilities. These findings as well as clinical and neurophysiological/neuroimaging findings are discussed below.

### Response Latencies

#### Fundamental Frequency Manipulation

Four *f*_0_ studies compared response latencies, the onset of the compensatory response to altered auditory feedback, across age groups and found that children consistently demonstrated longer response latencies to perturbations in auditory feedback than adult populations (Liu H. et al., 2010; Liu P. et al., 2010; Liu et al., 2013; Scheerer et al., 2013b). Two of these studies used multiple child age groups to explore potential age gradients within responses (Liu et al., 2013; Scheerer et al., 2013b). Scheerer et al. (2013b) found a main effect of age, where three of the four age groups under 18 (4–6, 7–10, and 11–13 years) independently demonstrated longer response latencies than the 18–30-year-olds (14–17-year-olds did not). Similarly, Liu et al. (2013) found their younger children (ages 10–12) had significantly longer response latencies than the adult group, however, older children (ages 13–15) did not differ from adults.

#### Response Latencies Summary

The finding of significantly longer response latencies for children compared to adults in the compensatory responses in pitch-shifted paradigms is consistent with developmental trends. Neurophysiological response latencies (i.e., event-related potential latencies) are considered an objective measure of the speed of neural integration and activity, reflecting the efficiency of information processing and the synaptic density in the auditory cortex, where shorter latencies reflect faster integration of auditory information (Eggermont, 1988; Kotecha et al., 2009). Vocal response latencies were similarly found to relate to maturational changes. Integration of rapid information in adult-like auditory processing may therefore be due to increased velocity and efficiency of neural conduction and intercortical communication in gray and white matter of the cortex.

### Response Magnitudes

#### Fundamental Frequency Manipulation

All *f*_0_ manipulation studies explored compensatory responses to pitch perturbations, and generally found children compensated in the opposite direction of the shift. Following responses were examined in three studies (Russo et al., 2008; Liu P. et al., 2010; Scheerer et al., 2020b), with two studies excluding participants who followed the perturbation (Scheerer et al., 2016; Demopoulos et al., 2018). Results exploring the magnitude of compensation responses to unexpected perturbations were mixed. When contrasting across age groups, children were found to have: (a) reduced magnitude responses compared to adults (Liu H. et al., 2010; Scheerer et al., 2016), (b) increased magnitude responses compared to adults (Liu P. et al., 2010; Heller Murray and Stepp, 2020), (c) increased responses that followed the manipulation compared to adults (i.e., following responses; Liu P. et al., 2010), and (d) no effect of age across childhood (Liu et al., 2013; Scheerer et al., 2013b,2020a) or compared to adults (Scheerer et al., 2013b; Heller Murray and Stepp, 2020). Heller Murray and Stepp (2020) found when analyzing opposing responses that only children with less sensitive pitch discrimination showed significantly larger responses, compared to adults and children with adult-like pitch discrimination. Liu et al. (2013) found an effect of sex, where male speakers produced larger response magnitudes than female speakers. Findings from Russo et al. (2008), Demopoulos et al. (2018), and Scheerer et al. (2020b) are described below in the *Clinical Findings* section.

The two studies exploring sustained perturbation found children showed smaller compensatory responses compared to adults (Scheerer et al., 2016; Heller Murray and Stepp, 2020). Specifically, Heller Murray and Stepp (2020) found children with less sensitive pitch discrimination produced smaller compensatory responses compared to adults and children with adult-like pitch discrimination. The magnitude of responses produced to a sustained shift was negatively correlated with the magnitude of responses to a sudden shift (Heller Murray and Stepp, 2020). In contrast, Scheerer et al. (2016) found that children produced smaller compensatory responses compared to adults in both sudden and sustained pitch shift conditions, however, these responses were not examined for correlation. Scheerer et al. (2016) also explored adaptation in the end phase, finding magnitudes of responses following removal of pitch perturbation did not differ between children and adults. In general, these findings provide evidence supporting the DIVA model, where responses to sudden and sustained shifts are not considered separate processes (Guenther, 2006; Guenther and Vladusich, 2012).

#### Formant Frequency Manipulation

All formant frequency manipulation studies explored compensatory responses, and overall found typically developing children generally showed compensation in the opposite direction of the perturbation in hold and end phases. Two studies examined following responses (Terband et al., 2014; van Brenk and Terband, 2020). Seven studies contrasted child and adult responses to formant manipulated altered auditory feedback (Shiller et al., 2010a; MacDonald et al., 2012; Daliri et al., 2018; Caudrelier et al., 2019; Kim et al., 2020; van Brenk and Terband, 2020; Ohashi and Ostry, 2021). Across the studies, children showed: (a) stronger compensation and adaptation responses in F1 than adults (van Brenk and Terband, 2020), (b) similar magnitude compensation to adults (Shiller et al., 2010a; MacDonald et al., 2012; Daliri et al., 2018; Kim et al., 2020; Ohashi and Ostry, 2021), and (c) no age effect in compensation or adaptation across childhood (Caudrelier et al., 2019). MacDonald et al. (2012) found young children showed similar compensation to adults, however, children under 4 years of age showed no compensatory response. Shiller and Rochon (2014) found after a period of perceptual training, children showed increased magnitude of compensation.

#### Response Magnitude Summary

Based on the underlying mechanisms being examined, response magnitudes for unexpected and sustained shifts were analyzed separately. Unexpected shifts, used to explore an individual’s reliance on auditory feedback control, examined in *f*_0_ manipulation studies, elicited mixed results, ranging from reduced magnitude to increased magnitude compensatory responses compared to adults to no age effect. One potential reason for these mixed findings could be due to proximity of shifted trials. As discussed in Cai et al. (2012), cross-trial adaptation effects have been found where participants’ early productions within a trial contain compensation responses to the perturbation of the previous trial.

By contrast, sustained shifts, used to explore the updating of feedforward control, generally showed no age effect after 4 years of age in formant manipulation studies. The lack of age effect suggests that children are using feedforward control similar to adults. In contrast, in *f*0 sustained shift studies, children exhibited smaller magnitude compensatory responses compared to adults. This smaller compensation response may indicate a greater reliance on sensory feedback, with reduced weighting on feedforward control.

Adaptive responses, when perturbations were removed, showed mixed results ranging from stronger adaptive responses compared to adults in formant manipulation studies, to no age effect across childhood in formant or *f*_0_ manipulation paradigms. Although mixed, these findings of the presence of adaptation responses show that children used the altered auditory feedback to update their sensorimotor mappings for future vocalizations.

Contrasting pitch and formant manipulation paradigms, clear developmental differences are seen in the youngest ages where children appear to be using their auditory feedback to manipulate their vocal productions. Scheerer et al. (2020a) found children as young as 2 years of age compensated to *f*_0_ shifted stimuli, whereas MacDonald et al. (2012) did not find compensatory responses in children under 4 years of age to formant shifted stimuli. This lack of compensation suggests that the ability to adaptively regulate measures of vocal control (i.e., *f*_0_) arises before control over measures of articulatory settings (i.e., formants). However, further research is required to confirm this assumption, as the number of studies examining responses in children under 3 years of age is restricted to these two studies.

### Vocal Variability and Perceptual Abilities

As variability in both the motor and perceptual system play an important role in feedback and feedforward control these correlates were examined together.

#### Fundamental Frequency Manipulation

Five studies explored differences in vocal variability in *f*_0_, contrasting baseline standard deviation in vocal productions across age groups (Russo et al., 2008; Scheerer et al., 2013b,^2016^, 2020a; Heller Murray and Stepp, 2020). Four of these studies contrasted children with adults, finding children consistently showed more variability than adults (Scheerer et al., 2013b,^2016^, 2020a). Heller Murray and Stepp (2020) found children with less sensitive pitch discrimination had significantly higher variability at baseline than both the children with adult-like pitch discrimination and adults. Children with less sensitive pitch discrimination also showed larger response magnitudes to unexpected pitch shifts and smaller responses to sustained pitch shifts compared with adults and children with adult-like pitch discrimination. Baseline vocal variability was also found to positively correlate with the magnitude of responses to unexpected perturbations, and negatively correlate with the magnitude of responses to sustained perturbations (Heller Murray and Stepp, 2020). Through regression analyses, Scheerer et al. (2013b) found that vocal variability accounted for a significant amount of the variance in the magnitude of the compensatory responses. Scheerer et al. (2016), however, did not find vocal variability correlated with the magnitude of compensatory responses. In further exploration of electrophysiological correlates, Scheerer et al. (2013b) found that age and vocal variability were significant predictors of N1 amplitude.

#### Formant Frequency Manipulation

Six studies explored variability of baseline vocal productions related to articulatory control (Shiller et al., 2010a; MacDonald et al., 2012; van den Bunt et al., 2018a; van Brenk and Terband, 2020; Coughler et al., 2021; Ohashi and Ostry, 2021). Generally, children showed greater variability compared to adults (Shiller et al., 2010a; MacDonald et al., 2012; van Brenk and Terband, 2020; Ohashi and Ostry, 2021). MacDonald et al. (2012) found that variability decreased with age. Similarly, van den Bunt et al. (2018a) found literate children who read more non-words per minute showed less variation in vowel production. Coughler et al. (2021) found increased variability negatively correlated with the amount of compensation in TD children, whereas Ohashi and Ostry (2021) did not find variability correlated with the amount of compensation in children or adults.

Five studies additionally examined perceptual abilities related to articulatory control (i.e., discrimination; Shiller et al., 2010a,b; Shiller and Rochon, 2014; Terband et al., 2014; Coughler et al., 2021). Coughler et al. (2021) found F1 discrimination thresholds did not significantly correlate with percent compensation in the positive or negative condition, or with language scores in TD children or children with a specific deficit in language. Shiller and Rochon (2014) found that productions following an experimental block of relevant (to the formant frequency shift) auditory-perceptual training resulted in enhanced compensatory responses in children. Based on results from a phoneme identification test, Shiller et al. (2010a) found children had more imprecise perceptual boundaries than adults. Additionally, while adults demonstrated a significant shift in their perceptual boundaries for the perturbed sound contrast after testing, children did not reliably change these internal perceptual boundaries.

#### Vocal Variability and Perceptual Abilities Summary

Studies involving *f*_0_ and formant manipulated feedback consistently showed increased variability in child baseline productions compared to adults. Individuals with more variable vocal productions are thought to have less defined internal sensorimotor representations. These sensorimotor representations encode the relationship of stored motor commands (utilized for feedforward control) and their auditory and somatosensory consequences (utilized for feedback control). Early in development, it is hypothesized, that children must rely more on auditory feedback during vocalization to ensure that their speech is in line with their desired vocal output, resulting in unstable vocal productions (Scheerer and Jones, 2012). As exposure to speech increases, the reliability of internal sensorimotor representations increases and over-dependence on auditory feedback becomes unnecessary, with vocal output becoming more consistent, shifting their reliance to feedforward control (Scheerer and Jones, 2012). Feedback control, however, continues to be an integral part of speech motor control, as auditory and somatosensory feedback are used to inform and maintain feedforward control, updating and refining sensorimotor representations when mismatches occur between expected and actual output (Franken et al., 2019).

Perceptually, findings across *f*_0_ and formant studies generally demonstrated reduced precision in vocal and articulatory control in children compared to adults. While baseline variability represents potentially different control mechanisms (i.e., articulatory or vocal), reduced perceptual discrimination, and increased vocal variability in younger children across paradigms aligns with the DIVA model where reliance is postulated to shift from feedback to feedforward control as sensorimotor targets are refined over multiple productions, with initial targets being larger and discrimination abilities less sensitive (Guenther, 2006; Tourville et al., 2008; Guenther and Vladusich, 2012). This was further supported by findings by MacDonald et al. (2012) where baseline F1 and F2 variability was found to decrease with age. This suggests that maturational changes occurring in the speech motor control system affect the extent to which auditory feedback is used to modify internal sensorimotor representations.

### Speech, Language and Literacy

Nine studies collected additional information related to speech, language, reading, cognitive, and social competence. Supplementary Material 4^3^ details the additional measures collected and related findings.

#### Speech and Language

Three studies collected articulation information, with two using the Goldman-Fristoe Test of Articulation 2 (GFTA-2; Shiller et al., 2010b; Daliri et al., 2018). Five studies collected receptive and expressive language information with the most common test used being the Clinical Evaluation of Language Fundamentals (CELF; Russo et al., 2008; Shiller et al., 2010b; Coughler et al., 2021). Five studies collected information about cognitive abilities with the most common test used being the Wechsler Abbreviated Scale of Intelligence (WASI; Russo et al., 2008; Daliri et al., 2018; Scheerer et al., 2020b; Coughler et al., 2021). In these studies results included: (a) no significant correlation found between speech and language tests and compensation (Daliri et al., 2018; Scheerer et al., 2020b; Coughler et al., 2021); (b) a significant correlation of response magnitudes with core, receptive and expressive language scores, where decreased magnitude responses were related to higher language scores (Russo et al., 2008); and (c) a significant positive correlation of compensation with performance on non-word repetition (Terband et al., 2014). Scheerer et al. (2020b) also found average response latency significantly predicted Multidimensional Social Competence Scale scores (MSCS).

#### Literacy

Phonological awareness measures were collected in three studies (van den Bunt et al., 2018a,b; Caudrelier et al., 2019). Reading measures were additionally collected in two of these studies (van den Bunt et al., 2018a,b). Better rapid automatized naming was found to correlate with better compensation (van den Bunt et al., 2018a), as well as correlated with weaker deviation from the baseline during the ramp-up phase and stronger de-adaptation during the ramp-down phase (van den Bunt et al., 2018b).

Average phonological awareness scores were significantly higher in children who compensated than to non-adapting children (Caudrelier et al., 2019), and was associated with stronger compensation during ramp-up and hold phase, and weaker de-adaptation in the ramp-down and end phases (van den Bunt et al., 2018b). Phonological awareness, rapid naming, and letter knowledge correlated significantly with compensation in preliterate children, whereas reading correlated with compensation in literate children (van den Bunt et al., 2018a). Overall, literacy was also found to play a role in compensatory response magnitude, where significantly stronger compensation in hold and end phases were found for literate children relative to preliterate children (van den Bunt et al., 2018a).

#### Speech, Language and Literacy Summary

In general, findings examining the relationship of speech, language, and cognitive measures in relation to compensation magnitude were limited. A few studies found no significant relationship of speech and language abilities with compensation, while one study found a significant negative relationship with language abilities. One possibility for these differences could be a result of differences in the assessment tools used to assess speech, and language. As well, although several studies collected information on speech, language and cognitive abilities, the relationship of these abilities with compensation magnitude were not examined.

A clear relationship however was evident for literacy in relation to the developmental trajectory of auditory feedback control. Reading and preliteracy skills (e.g., phonological awareness) significantly correlated with compensation ability. In particular, phonological awareness scores, a strong predictor of later reading ability, were significantly higher in children who compensated compared to those who showed no compensatory response (Caudrelier et al., 2019), and in those who showed greater compensation (van den Bunt et al., 2018b). These results suggest an interplay among auditory-integration, speech motor control, and reading, supporting theories that impaired phonological representations (essentially sensorimotor representations) may underlie reading deficiencies (Ramus and Szenkovits, 2008), although further exploration is needed to understand other potential factors that may influence this relationship.

### Clinical Findings

#### Fundamental Frequency Manipulation

Three studies exploring clinical population responses to *f*_0_ manipulated altered auditory feedback included children with autism spectrum disorder (ASD; Russo et al., 2008; Scheerer et al., 2020b) and children who are 16p11.2 deletion carriers (Demopoulos et al., 2018). Deletion at 16p11.2 is commonly observed in individuals with diagnoses of developmental coordination disorder, phonological processing disorder, language disorders, and ASD (Demopoulos et al., 2018). Russo et al. (2008) had mixed findings, where some children with ASD demonstrated smaller mean magnitude compensatory responses, while others created atypically large compensatory responses compared to TD children. Scheerer et al. (2020b) found children with ASD had shorter response latencies than TD children, but showed similar compensatory responses to TD peers. In contrast, Demopoulos et al. (2018) consistently found children with 16p11.2 deletion showed larger pitch compensation compared to controls.

#### Formant Frequency Manipulation

Seven studies explored compensatory responses to formant frequency manipulations of children with speech and language difficulties. Two of these studies examined children with SSD (Shiller et al., 2010b; Terband et al., 2014), two examined responses of children who stutter (Daliri et al., 2018; Kim et al., 2020), one of children with dyslexia (van den Bunt et al., 2018b), one with children who are 16p11.2 deletion carriers (Demopoulos et al., 2018), and one with children with developmental language disorders (DLD; Coughler et al., 2021). No consistent findings were seen across these clinical populations. Children who stutter were found in one study (Daliri et al., 2018) to show greater compensation in F1 than adults who stutter, however they did not differ from children who do not stutter. However, Kim et al. (2020) found children who stutter showed no compensatory response. Conflicting results were similarly found for children with SSD. Terband et al. (2014) found children with SSD followed the perturbation in F1 during hold and end phases, whereas Shiller et al. (2010b) found compensation as well as an adaptation response. It is important to note that Shiller et al. (2010b) only included one participant. Similar to Shiller et al. (2010b), van den Bunt et al. (2018b) found all children with dyslexia compensated in the opposite direction of the perturbation, with the only difference from typically reading peers being a weaker return to baseline during the ramp-down phase. In contrast, children with 16p11.2 deletion showed significantly weaker compensation than their TD peers (Demopoulos et al., 2018). Coughler et al. (2021) found a unique pattern where children with a specific deficit in language (DLD) demonstrated differential compensation in positive and negative shift conditions. Children with DLD showed larger compensation in the positive shift condition and compensated less in the negative shift condition compared to typically developing peers.

#### Clinical Findings Summary

Clinically, across formant and *f*_0_ manipulation studies, a broad range of disorder areas were examined, from ASD, 16p11.2 deletion, SSD, dyslexia, fluency, to DLD. All of these disorders have been linked to impairments in or closely linked to auditory processing. Although there was a lack of methodological consistency, several studies found aberrant responses in some of the clinical populations compared with typically developing children. This included increased following responses and larger or smaller compensation responses compared to typically developing peers.

Results involving children who stutter further support developmental sensorimotor control changes into adulthood. In Daliri et al. (2018), children who stutter produced greater compensation than adults who stuttered, however, they did not differ compared to children who do not stutter. This suggests some shift in reliance in adults, which is further supported by the finding that adults who stutter did not show any adaptation, whereas children who stutter did successfully adapt in their F1 productions. Adults who stutter were no longer updating their stored motor programs through feedforward control unlike children who stutter. Kim et al. (2020) found very different findings, children who stutter showed no significant compensatory response, although similarly, adults showed a reduced compensation compared to TD adults. Of note here, compensatory responses significantly correlated with age, where greater compensation occurred with increased age. Potential differences between these two studies may be due to differences in shifts applied, where Daliri et al. (2018) shifted the phonemic category of the vowels, and Kim et al. (2020) did not.

Neuroanatomically, the atypical mixed compensation responses seen in children with ASD in *f*_0_ manipulations studies (some in the typical range and others overcompensating) may relate to findings that children with ASD have weaker white matter connections between left ventral premotor cortex, a key area in speech motor planning, and other cortical regions involved in speech production (Russo et al., 2008; Peeva et al., 2013). Although not collected in their studies (Russo et al., 2008; Scheerer et al., 2020b), weaker white matter connections could be associated with the overcompensation profile found in some children. Scheerer et al. (2020b) additionally found shorter response latencies in children with autism, indicating more research is needed to further understand what may be driving differences.

Similarly, the mixed findings found for children with SSD, with some showing typical responses (Shiller et al., 2010b) and others showing increased following responses (Terband et al., 2014), could be related to gray and white matter volume differences. Previous studies have found abnormal gray and white matter volume in areas relating to speech motor control, which is thought to be related to delays in synaptic pruning (Preston et al., 2014).

Of interest, the type of manipulation was shown to affect the direction of aberrant responses. Demopoulos et al. (2018) found children who were carriers of 16p11.2 deletion showed overcompensation compared to controls in response to pitch perturbation (unexpected shift), but undercompensation compared to controls in response to formant manipulated feedback (sustained shift). This further supports theories that these vocal and articulatory controls develop at differing rates and time points. However, it is important to take into consideration that different experimental designs (i.e., sudden vs. sustained shift) may be the driving factor resulting in these differing compensation responses. Further research is needed contrasting responses to sudden and sustained shifts using consistent manipulations.

### Neurophysiological and Neuroimaging Findings

#### Fundamental Frequency Manipulation

Two studies explored EEG responses to *f*_0_ altered auditory feedback (Liu et al., 2013; Scheerer et al., 2013b). Both found that P1 and N1 latency, and P1 amplitude decreased with age (Liu et al., 2013; Scheerer et al., 2013b). Scheerer et al. (2013b) also found that N1 amplitude increased with age, however, Liu et al. (2013) did not find a significant effect of age on N1 amplitude. P2 amplitude showed greater variability, but generally was shown to increase with age (Liu et al., 2013; Scheerer et al., 2013b). Notably, Liu et al. (2013) also found significant interactions between sex and age in the N1 and P2 potentials. Male participants generally produced larger N1 amplitudes, and within the group of older children, females demonstrated significantly shorter N1 latencies than males. Among the young children, males had significantly larger P2 amplitudes than females, and young females had significantly larger P2 amplitudes than older females (Liu et al., 2013). In terms of P2 response latencies, P2 latency was found to be age-dependent for males only, however, within the group of older children, females were found to have significantly shorter P2 response latencies compared to males (Liu et al., 2013). The variable age-sex interactions found by Liu et al. (2013) speaks to the complexity of factors influencing neural responses to auditory feedback.

#### Formant Frequency Manipulation

Two studies (van den Bunt et al., 2018b; Ohashi and Ostry, 2021) examined neural activation in relation to formant manipulation sensorimotor control studies. van den Bunt et al. (2018b) used fractional anisotropy to measure connectivity of the arcuate fasciculus/superior longitudinal fasciculus (AF/SLF). Fractional anisotropy of the AF did not directly relate to altered auditory feedback responses, but did correlate strongly with phonological awareness scores. When phonological awareness was controlled, higher fractional anisotropy was found to be associated with less adaptation during altered auditory feedback (van den Bunt et al., 2018b). Ohashi and Ostry (2021) found children and adults had distinct patterns of functional connectivity. In adults, compensation to altered auditory feedback was positively correlated with activation in the right inferior frontal gyrus (area 44) and associative sensory regions. In children, compensation was positively correlated with functional connectivity of the primary somatosensory cortex (S1)/primary motor cortex (M1) and posterior rostral cingulate zone (RCZ) and left anterior insular cortex. When contrasting younger and older children (over 9 years), older children showed an increasingly adult-like pattern of connectivity.

#### Neurophysiological and Neuroimaging Findings Summary

Neurophysiological and neuroimaging studies involved examining evoked potentials, diffusion-weighted imaging and resting-state functional connectivity. Evoked potentials have a well-established history of being an objective measure of maturation of the nervous system, which can increase our understanding of neurophysiological changes that underlie behavioral responses to sensory input (Eggermont, 1988). Due to small sample sizes and differing languages (English in Scheerer et al., 2013b, and Mandarin in Liu et al., 2013), age-dependent conclusions are guarded, although both utilized similar *f*_0_ shifted paradigms. Developmental trends observed in P1-N1-P2 amplitudes and latencies mirrored general developmental trends found during passive listening tasks (Ponton et al., 2000; Wunderlich and Cone-Wesson, 2006; Fitzroy et al., 2015). These similarities support the existence of a developmental gradient in auditory integration.

The significant decreases in latency observed with age across both the P1 and the N1 components of the P1-N1-P2 complex (Liu et al., 2013; Scheerer et al., 2013b), alongside the decreases in behavioral response latencies, together provide significant support for the existence of age-related changes in the efficiency of auditory integration in the cortex. This in turn suggests that the efficiency of information processing in cortical areas supporting sensory function influences the developmental trajectory of speech motor control. Findings of consistent decreases in P1 amplitude (Liu et al., 2013; Scheerer et al., 2013b) and increases in N1 amplitude (Scheerer et al., 2013b) across age during the processing of altered auditory feedback provides more evidence for the age-dependent shift from reliance on feedback to reliance on feedforward control identified through the analysis of response magnitudes.

Neuroanatomically, although findings examining fractional anisotropy of the left arcuate fasciculus were not found to directly relate to compensation responses (van den Bunt et al., 2018b), resting-state functional connectivity showed distinct patterns of connectivity which significantly related to compensation in speech sensorimotor adaptation tasks (Ohashi and Ostry, 2021). This finding supports the hypothesis that protracted neural plasticity during development relates to differences in performance in speech motor learning, demonstrating that speech motor adaptation abilities relate to cortical remodeling and reorganization occurring across development.

## Summary

Speech motor control, in particular, auditory feedback is key to the development of speech, however, much remains unknown about how this develops in children. The current scoping review explored pediatric studies that examined frequency altered auditory feedback, with findings divided into fundamental and formant frequency manipulation studies. The aim of this scoping review was to gain a broad overview of the current state of research in pediatric frequency altered auditory feedback, investigating how responses to shifted auditory feedback change throughout development, thus expanding our understanding of the developing speech motor control system, and highlighting potential future directions and gaps in the literature. Searches from six academic databases retrieved twenty-three articles that explored various implementations of frequency manipulated altered auditory feedback. Results found age-dependent developmental trajectories related to response latencies, magnitude of compensatory responses, variability and perceptual abilities, as well as relationships of compensation with literacy.

### Age-Dependent Trajectory of Responses to Altered Auditory Feedback

The primary goal of this study was to gain a better understanding of how and when children use information from auditory feedback to regulate their speech. Results across both fundamental and formant frequency manipulated altered auditory feedback showed children above the age of four generally compensated for the altered auditory feedback in the opposite direction of the perturbation (MacDonald et al., 2012). This is consistent with previous research of pediatric responses to other forms of altered auditory feedback where children, like adults, adjusted their speech to perturbations of their vocal intensity, timing, and jaw/lip positioning (e.g., Chase et al., 1961; Siegel et al., 1976; Ménard et al., 2008). However, mixed findings across different measures were evident. For example, increased incidence of following responses, as well as larger and smaller compensatory responses in children compared to adults, suggests that pediatric populations may not be using auditory feedback for speech motor control in the same manner as adults. Results obtained using these different measures may provide key information about the developmental trajectory of auditory feedback control across childhood.

### Future Directions

Although the reviewed studies provided relevant findings about the potential of age-dependent changes in auditory feedback control, further research is needed. This scoping review found several limitations and gaps within the field, highlighting the need for further high quality quantitative well-designed studies.

The most significant limitation across these studies is a lack of power due to small sample sizes. Several of the studies discussed included around 20 participants, with only three studies including more than 30 participants in each group (Scheerer et al., 2013b,2020b; van den Bunt et al., 2018a). Some studies also failed to report effect sizes, making power computations not possible. Creating well-powered studies, with consistent reporting of effect sizes, would enable a more expansive systematic or meta-analysis in the future.

In terms of age ranges explored, very few studies of *f*_0_ manipulation explored younger ages (primarily focusing on school age), whereas formant manipulation studies explored a broader range. Scheerer et al. (2013b) and Scheerer et al. (2020b) were the only studies to examine a broad age range, looking at children 4–17 (as well as adults 18–30 years) and 3–13 years, respectively, in pitch perturbation. A more expansive look at changes across development is particularly missing in formant manipulations studies. Expanding age ranges within studies, examining changes across childhood, between young and older children, would provide clearer information about developmental changes in responses. Additionally, utilizing longitudinal studies may clarify maturational changes, taking into account the increased variability found in younger children.

In light of the several other subsystems developing in parallel with speech motor control, more comprehensive data collection is necessary. While several of the studies discussed in this scoping review examined aspects of other systems (e.g., clinical measures, neurophysiological, and perceptual), no study provided a comprehensive examination, combining information about neural processing, and parallel skill development (e.g., speech, language, and literacy) in relation to behavioral performance (e.g., vocal response magnitude).

Overall, gaps in the literature highlight the need for more comprehensive, larger sample, broad age range studies, with multiple outcome measures (e.g., magnitude, response latency, language, phonological awareness, literacy, and auditory perception).

## Conclusion

The current scoping review provides a detailed description of the current state of research on pediatric responses to conditions of *f*_0_ and formant shifted altered auditory feedback, and highlights critical gaps in the literature. As discovered, only a small body of research exists to date that addresses pediatric responses to frequency altered auditory feedback. Within the 23 articles reviewed, significant variability was seen in methodological frameworks, manipulations applied, as well as languages of participants, and age ranges. Significant variability in the characteristics of behavioral responses across these studies also leads to difficulties in generalizing and identifying age-dependent trends.

While this review provides key information about age-related changes in auditory integration and the development of speech motor control, there is a pressing need for future research in this area in order to understand further the general cognitive development of speech motor control.

## Data Availability Statement

The original contributions presented in the study are included in the article/Supplementary Material, further inquiries can be directed to the corresponding author/s.

## Author Contributions

KQdL and DB contributed to the initial conception and design of the study. KQdL created initial search terms, conducted the initial literature review, and wrote the first draft of the manuscript. CC updated search terms and literature review and updated the manuscript. All authors contributed to editing the manuscript, and approved the submitted version.

## Conflict of Interest

The authors declare that the research was conducted in the absence of any commercial or financial relationships that could be construed as a potential conflict of interest.

## Publisher’s Note

All claims expressed in this article are solely those of the authors and do not necessarily represent those of their affiliated organizations, or those of the publisher, the editors and the reviewers. Any product that may be evaluated in this article, or claim that may be made by its manufacturer, is not guaranteed or endorsed by the publisher.

## Footnotes

1
https://osf.io/p9jz8/

2 One study included both fundamental and formant frequency manipulations.

3
https://osf.io/2jwt4/
