# Supplementary material for: Pediatric Responses to Fundamental and Formant Frequency Altered Auditory Feedback: A Scoping Review
Source: Front Hum Neurosci. 2022 May 17;16:858863. doi: 10.3389/fnhum.2022.858863 (PMC9157279; doi:10.3389/fnhum.2022.858863)

Supplementary 1: Search term strategy

1. Keyword searches

| **#** | **Keyword search terms** |
| --- | --- |
|  | **Frequency altered auditory feedback keywords** |
|  | *Note.* Adjacency term modified based on database:  CINAHL: N#  Embase and Medline: adj#  PsycInfo and Web of Science: NEAR/#  Scopus: W/# |
| 1 | Alter* adj3 “auditor* feedback” |
| 2 | “Auditor* perturbation*” |
| 3 | Auditor* adj3 “feedback perturbation*” |
| 4 | Formant adj3 perturbation* |
| 5 | Formant adj3 shift* |
| 6 | Formant adj3 compensat* |
| 7 | “Speech motor” adj3 learning |
| 8 | “Speech motor” adj3 adaptat* |
| 9 | “Auditory motor” adj3 learning |
| 10 | Pitch adj3 “feedback perturbation*” |
| 11 | Perturb* adj3 “auditory feedback” |
| 12 | “Articulator* compensat*” |
| 13 | Auditor* adj3 “feedback control” |
| 14 | Alter* adj3 “fundamental frequency” |
| 15 | “Fundamental frequency” adj3 manipulat* |
| 16 | Frequenc* adj3 “alter* feedback” |
| 17 | Frequenc* adj3 “shift* feedback” |
| 18 | Frequenc* adj3 “alter* auditor* feedback” |
| 19 | 1 OR 2 OR 3 OR 4 OR 5 OR 6 OR 7 OR 8 OR 9 OR 10 OR 11 OR 12 OR 13 OR 14 OR 15 OR 16 OR 17 OR 18 |
|  | |
|  | **Pediatric keywords** |
|  | *Note.* Adjacency term modified based on database:  CINAHL: W#  Embase and Medline: adj#  PsycInfo and Scopus: PRE/#  Web of Science: NEAR/# |
| 20 | Child* |
| 21 | Teen* |
| 22 | Young person* |
| 23 | Young people |
| 24 | Youth* |
| 25 | Pre-schooler* |
| 26 | Preschooler* |
| 27 | Pre adj2 school |
| 28 | School adj2 age* |
| 29 | Schoolage* |
| 30 | Kid* |
| 31 | Toddler* |
| 32 | Juvenil* |
| 33 | Pubescen* |
| 34 | Kindergar* |
| 35 | Girl |
| 36 | Boy |
| 37 | P?ediatric* |
| 38 | Adolescen* |
| 39 | 20 OR 21 OR 22 OR 23 OR 24 OR 25 OR 26 OR 27 OR 28 OR 29 OR 30 OR 31 OR 32 OR 33 OR 34 OR 35 OR 36 OR 37 OR 38 |
|  | 19 AND 39 |

1. MeSH search terms

| **Database** | **Frequency altered auditory feedback MeSH terms** | **Pediatric MeSH terms** |
| --- | --- | --- |
| CINAHL | sensory motor integration | child development  adolescence  child |
| Embase | auditory feedback  sensorimotor integration  motor learning | child development  adolescent  child |
| Medline (Ovid) | feedback, sensory | child development  adolescent  child |
| PsycInfo (Proquest) | auditory feedback | childhood development |

Supplementary 2: Sample search from PsycInfo (Proquest)


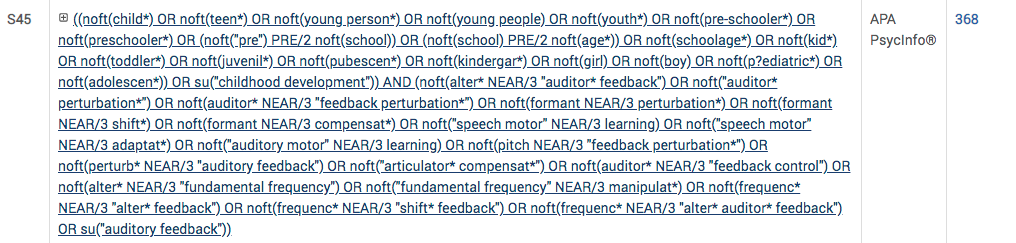

Supplement: Supplementary file 1 [file Data_Sheet_1.docx]
